# Identification of prognostic biomarkers associated with tumor microenvironment in ceRNA network for esophageal squamous cell carcinoma: a bioinformatics study based on TCGA database

**DOI:** 10.1007/s12672-021-00442-5

**Published:** 2021-11-01

**Authors:** Danlei Song, Yongjian Wei, Yuping Hu, Xia Chen, Ya Zheng, Min Liu, Yuping Wang, Yongning Zhou

**Affiliations:** 1 The First Clinical Medical College, Lanzhou University, Lanzhou, China; 2 Department of Gastroenterology, The First Hospital of Lanzhou University, Lanzhou, 730000 China; 3 Key Laboratory for Gastrointestinal Diseases of Gansu Province, The First Hospital of Lanzhou University, Lanzhou, 730000 China; 4 The First Department of Hepatobiliary and Pancreatic Surgery, Cangzhou Central Hospital, Cangzhou, China; 5 Hospital of Reproductive Medicine, The First Hospital of Lanzhou University, Lanzhou, China

**Keywords:** Esophageal squamous cell carcinoma, Tumor microenvironment, Weighted gene co-expression network analysis, Competing endogenous RNA network, Prognosis, Relative abundance of tumor infiltrating immune cells

## Abstract

**Background:**

Esophageal squamous cell carcinoma (ESCC) is the most common histological type of esophageal cancer in the world with high incidence rate and poor prognosis. Infiltrated immune and stromal cells are vital components of tumor microenvironment (TME) and have a significant impact on the progression of ESCC. The competitive endogenous RNA (ceRNA) hypothesis has been proved important in the molecular biological mechanisms of tumor development. However, there are few studies on the relationship between ceRNA and ESCC TME.

**Methods:**

The proportion of tumor-infiltrating immune cells and the amount of stromal and immune cells in ESCC cases were calculated from The Cancer Genome Atlas database using the CIBERSORT and ESTIMATE calculation methods. After stratified identification of differentially expressed genes, WGCNA and miRNA prediction system were applied to construct ceRNA network. Finally, PPI network and survival analysis were selected to discriminate prognostic signature. And the results were verified in two independent groups from Gene Expression Omnibus and Lanzhou, China.

**Results:**

We found that high Stromal and ESTIMATE scores were significantly associated with poor overall survival. Three TME-related key prognostic genes were screened, namely, LCP2, CD86, SLA. And the expression of them was significantly correlated with infiltrated immunocytes. It is also found that ESTIMATE Score and the expression of CD86 were both related to TNM system of ESCC.

**Conclusions:**

We identified three novel TME-related prognostic markers and their lncRNA-miRNA-mRNA pathway in ESCC patients, which may provide new strategies for the targeted therapy.

**Supplementary Information:**

The online version contains supplementary material available at 10.1007/s12672-021-00442-5.

## Introduction

Esophageal cancer, the 7th most common malignancy in the world, is also the sixth leading cause of cancer death, with a five-year survival rate of less than 20% and approximately 70% of cases occurring in men [1, 2]. Esophageal cancer is mainly divided into two types that differ in epidemiology and pathology: esophageal squamous cell carcinoma (ESCC) and esophageal adenocarcinoma (EAC). ESCC accounts for 80% of the global esophageal cancer cases, especially in China, which accounts for 90–95% [3]. Surgical treatment combined with chemotherapy and radiation therapy is the standard treatment for ESCC. However, the survival rate of patients remains unsatisfactory due to its high invasiveness and metastasis nature. New therapies tailored to molecular composition of the tumor are urgently required in order to improve prognosis.

Tumor microenvironment (TME) is the cellular environment in which tumor cells or tumor stem cells exist [4]. It consists of cell components, blood vessels, extracellular matrix ECM and various signaling molecules [5]. TME plays a major role in maintaining the activity of tumor stem cells, promoting tumor cell proliferation and facilitating neoplasm metastasis [6]. Immune cells are integral part of TME. Currently, the treatments associated with TME were principally targeted adaptive immune T cells, such as immune checkpoint blockade and chimeric antigen receptor T cell immunotherapy. Innate immune component cells can affect TME indirectly by controlling T cells, and even regulate TME precisely. For example, tumor associated macrophages, accounting for 50% of some solid tumors, can exert both pro-inflammatory effects to kill tumor cells and immunosuppressive effects to promote disease progression and resistance to therapy [7]. Ongoing clinical trials targeting macrophage receptor, CSF-1R and CCL2-CCR2 signal transduction axis have displayed therapeutic prospect in advanced solid tumors. Therefore, the thought of stimulating pro-inflammatory innate immune cells to improve TME provides a new direction for tumor treatment. Searching specific targeting marker is critical to reactivate antitumor immune response.

One RNA transcript can regulate the expression of another by isolating shared miRNAs. This complex interaction between RNA species is known as ceRNA crosstalk [8]. This mechanism is widely existed in the regulation of oncogenes and anti-oncogenes during tumorigenesis [9] and angiogenesis during development [10]. LncRNA-miRNA-mRNA, the most common ceRNA crosstalk, is widely applied in establishing tumor prediction model of prognosis [11, 12]. Current ceRNA studies of ESCC are generally focused on the differential genes identified between ESCC and normal tissues, but no known module about ceRNA network based on TME of ESCC has been constructed.

Transcriptome-sequencing patterns along with functional genomics analysis have elucidated the function of different types of cells during TME regulation. In our article, we utilized ESTIMATE and CIBERSORT computational methods to evaluated TIC proportion and the ratio of immune and stromal components of ESCC patients from TCGA database. Differentially expressed lncRNAs, miRNAs and mRNAs were screened with ESTIMATE Score system. And the WGCNA was applied to identify the modules most associated to the ESCC TME. Subsequently, we constructed the immune-related lncRNA-miRNA-mRNA ceRNA network. Ultimately three predictive biomarkers were distinguished consisting of LCP2, SLA and CD86. This finding contributes to the understanding of the role of TME in ESCC and explains the occurrence as well as development of ESCC.

## Methods

### Data acquisition

We downloaded gene expression profiles including lncRNA, miRNA and mRNA detection data from The Cancer Genome Atlas database^1^ (TCGA), the clinical information was also obtained. The inclusion criteria of this study was: (1) cancers confirmed by pathology, (2) primary tumor, (3) non-paraneoplastic tissue samples, (4) possessing complete clinical information, (5) removing patients with special histological types. We selected the cases with a histological type of squamous carcinoma and finally obtained information of 94 samples. The Estimate algorithm can use gene expression data to predict stromal and immune cells infiltrated in tumor tissue [13]. We obtained estimated values for these 94 samples using the estimate package (Version 1.0.13). For further verification, we also downloaded another group of 179 ESCC patients’ information from the Gene Expression Omnibus^2^ (GEO) database (GSE53625).

### Screen of differentially expressed genes (DEGs) and functional enrichment analysis

The gene expression data of 94 ESCC samples were filtered as follows: (1) removing the samples with incomplete lncRNA, miRNA or mRNA expression profiles; (2) discarding the genes with more than half of the samples with zero expression. Eventually, 80 patients were screened out. The median value of TME-related scores were used for stratification and the Limma package (version 3.44.3) was used for analysis of gene differential expression [14]. All q values used FDR to correct the statistical significance of the multiple tests. The lncRNAs and mRNAs with |logFC|> 1.2 and FDR < 0.05 were considered significant while the miRNAs with |logFC|> 1 and FDR < 0.05 were considered significant. Then all DEGs were input into R (version 4.0.2) for clustering analysis. The results were visualized by the pheatmap package. Each column represents a sample and each row represents the expression level of a given gene. After that, we used The Database for Annotation, Visualization and Integrated Discovery^3^ (DAVID) database to analyze DEGs’ abundant biological functions including Gene Ontology analysis (GO) and Kyoto Encyclopedia of Genes and Genomes (KEGG) analysis. The Go analysis is divided into three parts of biological process (BP), Molecular Function (MF) and cellular component (CC). FDR < 0.05 was statistically significant.

### Weighted gene co-expression network analysis (WGCNA)

WGCNA is a systematic biological method to describe gene association patterns in microarray samples. In our study, R package WGCNA (Version:1.69) was used to construct a gene co-expression network of mRNA/lncRNA high-throughput expression profile [15]. Co-expression relationships between adjacency matrices were assessed by Pairwise Pearson correlation analysis and a hierarchical clustering tree was drawn, with different branches of the tree representing different gene modules. The method of dynamic tree cutting was used to generate co-expression modules. Then the correlation between each module and clinical phenotype was calculated to obtain the closet lncRNAs and mRNAs relevant to ESCC tumor microenvironment. Finally, GO and KEGG were used to analyze the biological functions of the modules that were highly related to specific clinical features with a cut-off value of FDR < 0.05.

### CeRNA network construction

All LncRNA and mRNA were utilized to construct the ceRNA network in the most relevant module of WGCNA, and so were the differentially expressed miRNAs. Firstly, we used miRcode^4^ and Diana Tools^5^ databases to search targeted regulation between lncRNAs and miRNAs. Then miRTarBase,^6^ miRDB^7^ and Targetscan^8^ were consulted to predict target mRNA that miRNA may regulate. The ceRNA network was finally constructed according to the negative regulatory target relationships between miRNA-mRNA and miRNA-lncRNA.

After the ceRNA network was completed, we used the Search tool for the retrieval of interacting genes database V11^9^ (String) [16] to explore the interaction between mRNAs identified by the ceRNA network as well as construct protein–protein interaction (PPI) network. The interaction pairs with medium confidence which minimum required interaction score was 0.4 were considered meaningful and reserved. Then the PPI network is imported into the visualization software Cytoscape (version 3.8.2) [17]. The importance of these genes in PPI network was explored using the cytohubba plugin [18], and the screened genes were ranked and visualized according to their scores.

### Screening for key genes associated with survival in ESCC patients

Kaplan–Meier plots were drawn using the survival package (Version 3.2–3) in R to assess the association between lncRNA, miRNA, and mRNA expression levels in the ceRNA network and overall survival of ESCC patients in the PPI network. The association was tested by log-rank test. Univariate and multivariate Cox regression assessments were used to further analyze the effect of clinical parameters and gene expression levels on the survival of ESCC patients. P < 0.05 was considered statistically significant.

### Gene set enrichment analysis (GSEA)

Using GSEA method, we further explored the functional profile of core genes. The target sets used in GSEA were gene set c2 (cp.kegg.v7.0.symbols.gmt) from the Molecular Signatures Database (MSigDB)^10^ [19]. It was derived from published articles and online databases. In the GSEA software [20] (version 4.1.0), we compared the different biological functions and pathways enriched by high and low expression groups of core genes. It was considered that P < 0.05 and FDR < 0.25 had statistical significance.

### Relative abundance of tumor infiltrating immune cells (TICs)

CIBERSORT^11^ is the tool to calculate cell composition in tumor tissues with gene expression profile where the LM22 gene file can be used to define 22 immune cell subsets [21]. We estimated the TIC abundance profile of 80 subjects from TCGA database. Analyses of diversity and correlation between critical prognostic genes and TIC proportion were also executed. P < 0.05 was considered to be statistically significant.

### Immunohistochemistry (IHC) and H&E staining

As CD86 is closely related to the survival and clinical parameters for ESCC patients, we chose it to further validate the results. A total of 27 paraffin-embedded tissue specimens from patients who had radical operations at the Lanzhou University First Affiliated Hospital, from 2004 to 2019, were collected. The demographics of patients are presented in Supplementary S2. Tissue samples were cut into 4 um serial sections, and at least one out of the sections was available to identify stromal. IHC and H&E Staining were performed as Supplementary S3. All slides were analyzed by inverted microscope (CKX41, OLYMPUS; Japan). The result of IHC was evaluated with Image Pro plus. We counted the number of nucleated and stained cells per field and changed into density as cells/mm2 to estimate the expression level of CD86.Two pathologists who were blinded to clinical data respective evaluated three areas at × 200 magnification and the mean value was adopted.

## Results

### Stromal and ESTIMATE Scores are associated with ESCC clinical features

We obtained gene expression profiles and clinical features of 94 patients from TCGA database (Supplementary S1). Among them, 81 were males (86.2%) and 13 were females (13.8%), with a median age 57 years old (range 36–90). The median Stromal Score was − 465.1 (range − 1864.28  to 1300.98), the median Immune Score was − 97.31 (range − 1392.55 to  3397.88), and the median ESTIMATE Score was − 572.14 (range − 2679.92 to 3971.34).

**Fig. 1:**
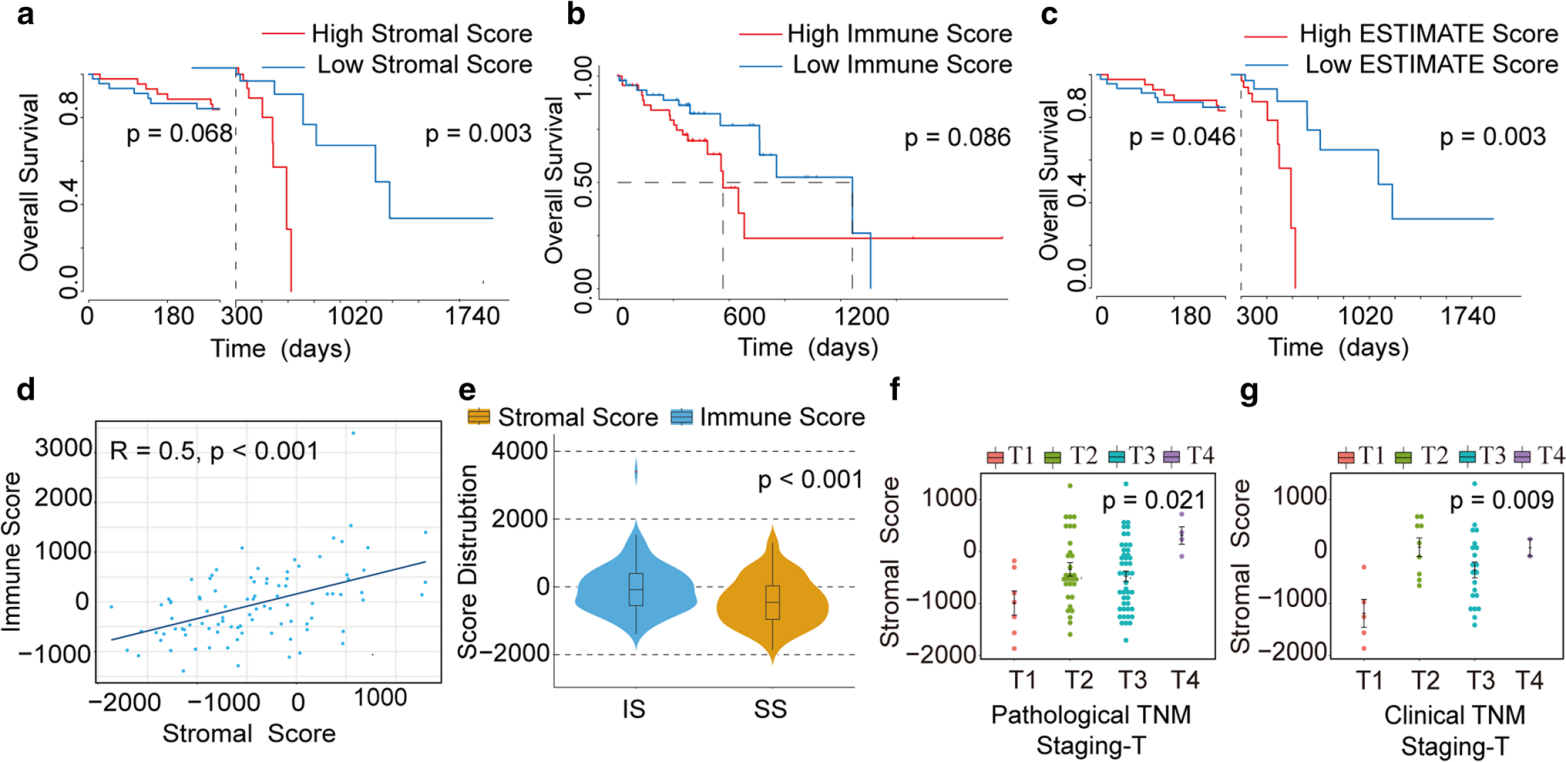
Relationship between scores and clinical parameters of patients with ESCC. **a** Kaplan–Meier survival curve of landmark analysis between Stromal Score and overall survival of patients with ESCC. **b** Survival analysis with Kaplan–Meier method for ESCC patients grouped by Immune Score (Log-rank test). **c** Kaplan–Meier survival curve of landmark analysis between ESTIMATE Score and overall survival of patients with ESCC. **d** The analysis of correlation between Stromal Score and Immune Score (Person analysis). **e** The analysis of diversity between Stromal Score and Immune Score (Wilcoxon rank sum test). **f**–**g** Distribution of Stromal scores in T classification

Higher scores of Immune Score or Stromal Score indicate more immune or stromal components in the TME. ESTIMATE Score, the sum of Immune and Stromal scores, indicates the comprehensive proportion of both components in TME. To evaluate the correlation between the proportion of immune or stromal components and the overall survival rate, ESCC patients were divided into a low immune/Stromal Score group and a high immune/Stromal Score group using the median immune or Stromal Score as the threshold. And survival analysis was performed using the SURVIVAL package (Version 3.2–3). Although Stromal Score (P = 0.068; Fig. 1a) and Immune Score (P = 0.086; Fig. 1b) weren't significantly related to survival, there was a significant difference in overall survival between patients with high ESTIMATE score and low ESTIMATE score (P = 0.046; Fig. 1c).

As the Kaplan–Meier survival curves of Stromal and ESTIMATE Scores intersected at nearly 1 year, we carried out landmark analysis with one year as the critical point. The results showed that after one year, the survival rate of patients with low Stromal Score was significantly higher than that of patients with high Stromal Score (P = 0.003; Fig. 1a), and the same results were demonstrated in ESTIMATE Score (P = 0.003; Fig. 1c). These results implied that Stromal and ESTIMATE Scores were more suitable for indicating the prognosis of ESCC patients.

Although the difference was not significant, the survival curve of Immune Score had the same trend as that of Stromal Score. Using Person method, we analyzed the correlation between Immune Score and Stromal score. The result showed the positive correlation between them (r = 0.05, P < 0.001; Fig. 1d). While Wilcoxon rank sum test also indicated the significant difference between them (P < 0.001; Fig. 1e). These results implied that stromal components and immune components are related but independent of each other in TME. In addition, we further explored the correlation between Stromal/Immune/ESTIMATE Scores and ESCC clinical parameters. Stromal Score had strong correlations with pathological/clinical TNM staging-T. The rank order of Stroma Score of ESCC pathological TNM staging-T was T4 > T2 > T3 > T1 (P = 0.021; Fig. 1f), and the Stromal Score of ESCC clinical TNM staging-T was T2 > T4 > T3 > T1 (P = 0.009; Fig. 1g).

### Identification of differentially expressed genes (DEGs) based on Stromal and ESTIMATE Scores

**Fig. 2:**
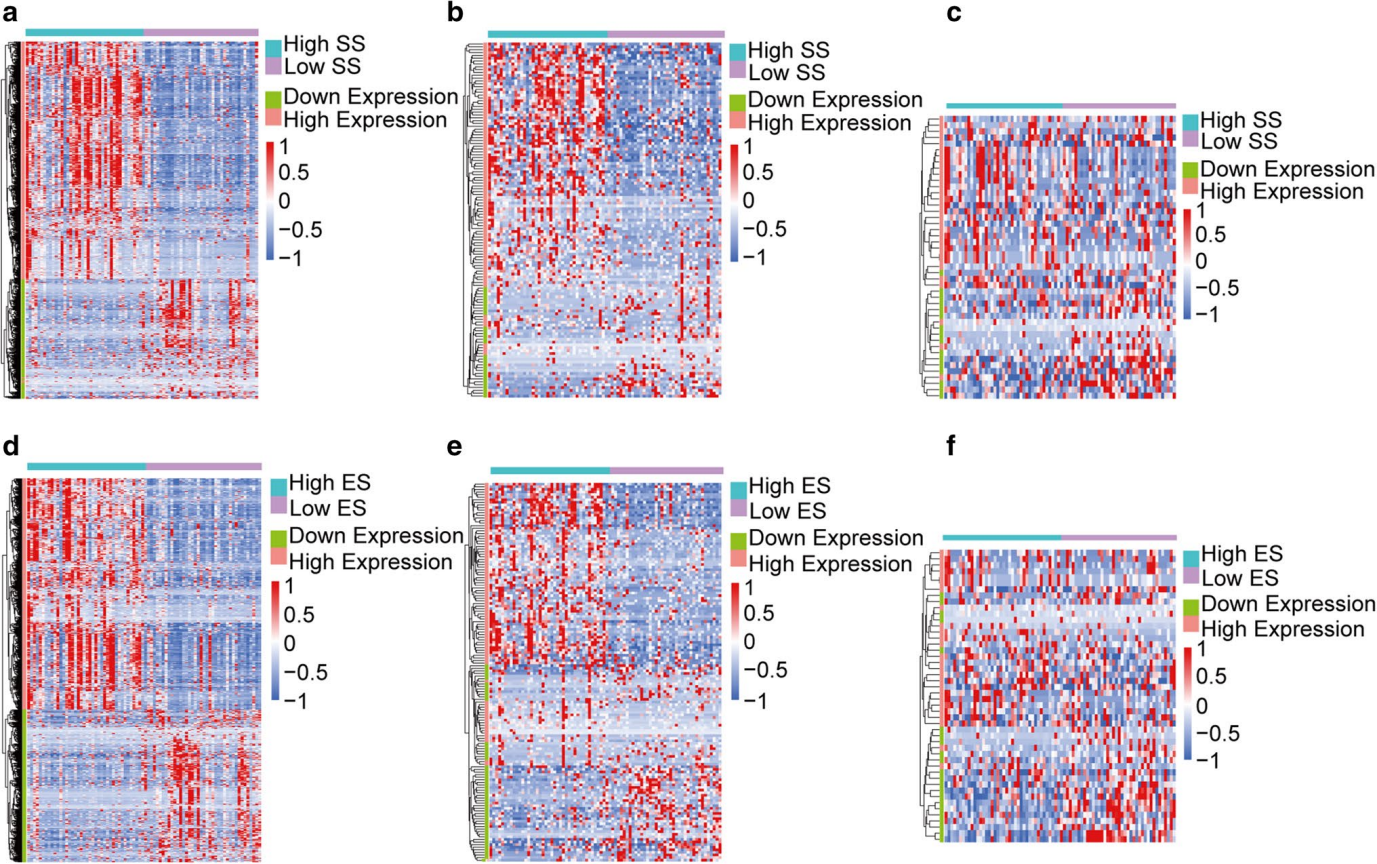
Heatmap of differentially expressed genes based on Stromal/ESTIMATE Score.**a**–**c** Differentially expressed mRNAs, lncRNAs, miRNAs s in the high and low Stromal Score groups. **d**–**f** Differentially expressed mRNAs, lncRNAs, miRNAs s in the high and low ESTIMATE Score groups

Given the results of survival analysis, we chose Stromal and ESTIMATE Scores for further analysis. Setting the cut-off criteria as | log FC |> 1.2 and FDR < 0.05, we identified 685 mRNAs (Fig. 2a) and 125 lncRNAs (Fig. 2b) based on Stromal Score, and 656 mRNAs (Fig. 2d) and 145 lncRNAs (Fig. 2e) based on ESTIMATE Score. Setting the cut-off criteria as |log FC|> 1 and FDR < 0.05, we identified 46 and 48 miRNAs based on Stromal Score (Fig. 2c) and ESTIMATE Score (Fig. 2f).

**Fig. 3:**
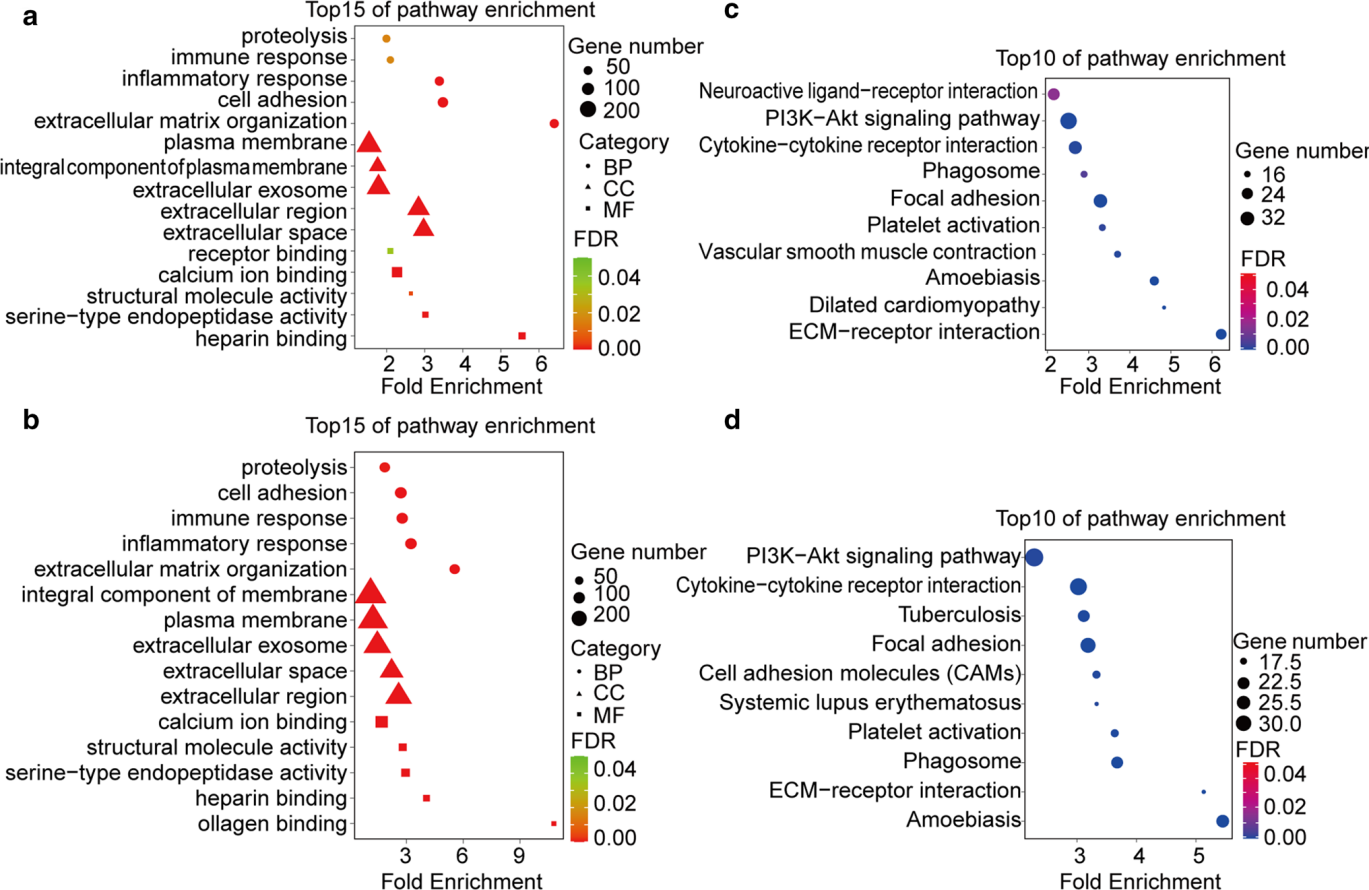
Functional enrichment clustering analysis for DEGs. **a**, **b** Top15 significant GO terms based on Stromal/ESTIMATE Score. **c**, **d** Top 10 KEGG terms based on Stromal/ESTIMATE Score

To comprehend the potential function of DEGs we used David gene annotation tool for performing GO annotation, and top 5 GO terms in BP, CC and MF were shown. The figures showed that DEGs based on Stromal and ESTIMATE Score almost mapped to proteolysis terms and stromal-related terms such as extracellular matrix organization and cell adhesion. Besides, some genes were enriched in immune and inflammatory response (Fig. 3a, b). Moreover, we performed KEGG signaling pathway enrichment analysis and top 10 KEGG terms showed that the genes based on Stromal and ESTIMATE Scores were both concentrated in extracellular matrix related pathway, including Cytokine-cytokine receptor interaction, Focal adhesion, ECM-receptor interaction and PI3K-Akt signaling pathway. (Fig. 3c, d). These results indicated that stromal components played an important role in ESCC TME. Certainly, the combined effect of stromal and immune components can’t be ignored.

### Construction of WGCNA network and identification of key module

**Fig. 4:**
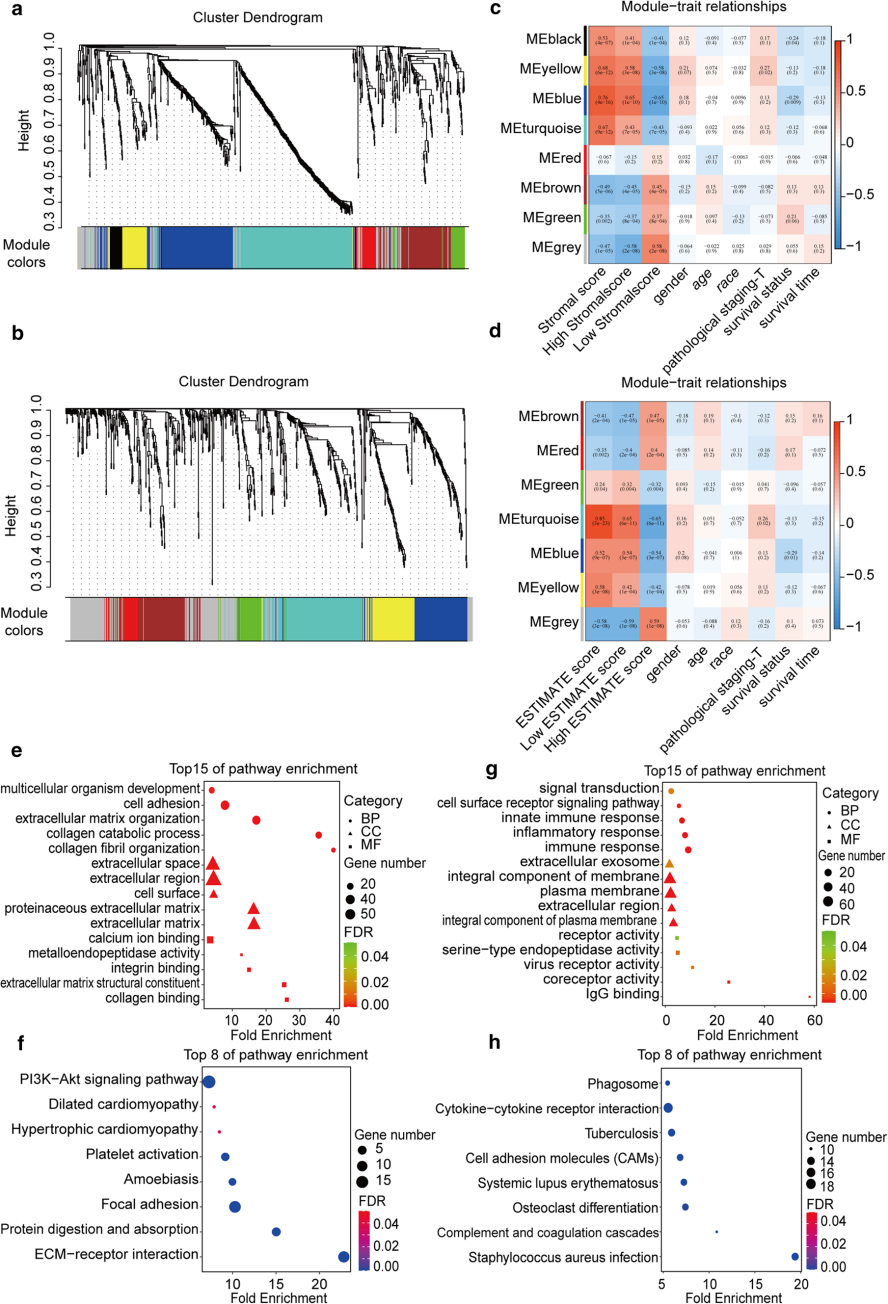
Clustering and module identification of differentially expressed mRNAs/lncRNAs using WGCNA. **a–b** Hierarchical clustering dendrogram based on the dissimilarity between DEmRNAs and DElncRNAs related to Stromal Score/ESTIMATE Score. The colored rows indicate modules with highly interconnected genes. Characteristics. **c–d** the relationship between features and module was calculated using the connection of genes in modules based on Stromal/ESTIMATE Score and their corresponding clinical characteristics. **e** Top 15 significant GO terms of blue module based on Stromal Score. **f** Top 15 significant GO terms of turquoise module based on ESTIMATE Score. **g** Top 8 KEGG terms of blue module based on Stromal Score. **h** Top 8 KEGG terms of turquoise module based on ESTIMATE Score

To find the key module related to ESCC TME, we chose DEGs based on Stromal and ESTIMATE scores for WGCNA analysis. Firstly, the samples were analyzed with hierarchical cluster and the outliers were removed. As the soft-threshold power parameter, β enhances the strong correlation and penalizes the weak correlation between genes. The β parameters of lncRNA and mRNA expression profile based on Stromal and ESTIMATE were 4 and 8, respectively. The method of dynamic tree cutting is used to identify co-expression modules. We ultimately generated 7 modules based on Stromal Score and 6 modules based on ESTIMATE Score. The results were displayed by the heatmap of topological overlap matrix (TOM). (Fig. 4a, b). WGCNA transformed adjacent matrix into the TOM, and further turned the association between different phenotypes into the correction between phenotype and gene sets. Moreover, we estimated the relevance between modules and clinical characters (Fig. 4c, d). These clinical features contained Stromal and ESTIMATE Scores, gender, age, race, pathological TNM stage, T stage, survival status and survival time. Blue module displayed the highest correlation with ESCC Stromal Score (r = 0.76), which included 161 mRNAs and 22 lncRNAs (Fig. 4c); and turquoise module displayed the highest correlation with ESCC ESTIMATE Score (r = 0.85), which included 159 mRNAs and 24 lncRNAs (Fig. 4d). Then we further used these 161 and 159 mRNAs for gene enrichment analysis. The results demonstrated that Stromal Score related genes were concentrated on Stromal component related process including extracellular matrix organization, extracellular matrix and extracellular matrix structural constituent (Fig. 4e). KEGG pathway analysis showed that these genes were mainly enriched in PI3K-Akt signaling pathway, platelet activation, collagen adhesion and ECM receptor interaction (Fig. 4f). On the other side, ESTIMATE related genes were most related to signal transduction, immune response, integral component of plasma membrane and IgG binding (Fig. 4g). In addition, genes were highly enriched in complement and coagulation cascades, osteoclast differentiation, cell adhesion molecules and cytokine-cytokine receptor interaction by KEGG analysis (Fig. 4h).

### CeRNA and PPI network construction

**Fig. 5:**
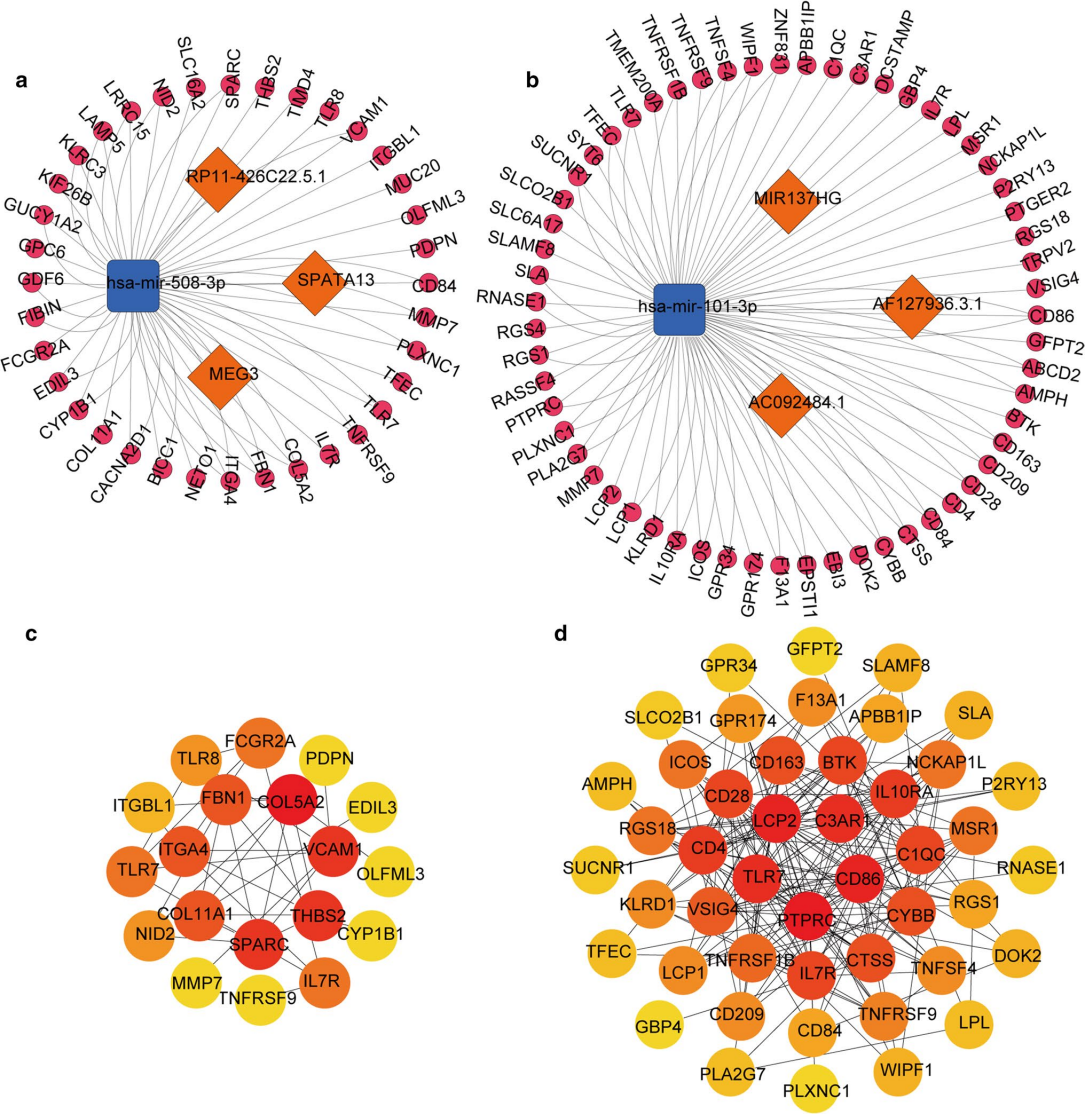
Construction of ceRNA interaction network and PPI network. **a**, **b** CeRNA network based on Stromal/ESTIMATE Score. The orange diamond represents the up-regulated lncRNA, the blue rounded rectangle represents the down-regulated miRNA, and the rose red circle represents the up-regulated mRNA. **c**, **d** PPI network of key mRNA based on Stromal/ESTIMATE Score constructed by DAVID database. Node represents DEmRNAs (confidence score > 0.4) and the color of each node represents the degree score of each node

On the basis of WGCNA results, we selected lncRNAs and mRNAs in Stromal Score related blue module and 46 differentially expressed miRNAs based on Stromal Score to construct the ceRNA network. And another network was composed of lncRNAs and mRNAs in ESTIMATE Score related turquoise module and 48 differentially miRNAs based on ESTIMATE Score. Using Diana Tools and miRBase online database, 14 lncRNA-miRNA pairs on Stromal Score and 5 lncRNA-miRNA pairs on ESTIMATE Score were matched. And 58,074 and 41,456 miRNA-mRNA pairs based on Stromal Score and ESTIMATE Score were searched by miRTarBase, TargetScan and miRDB online databases. Subsequently, the predicted lncRNAs and mRNAs were intersected with the genes in selected module. Finally, we plotted ceRNA networks associated with Stromal and ESTIMATE Scores by integrating the miRNA-lncRNA-mRNA interactions (Fig. 5a, b). The ceRNA network based on Stromal Score comprised 39 nodes (3 lncRNAs, 1 miRNA, 35 mRNAs) and 38 edges and another network base on ESTIMATE Score consist of 63 nodes (3 lncRNAs, 1 miRNA, 59 mRNAs) and 62 edges. We also discovered that the regulatory relationship among lncRNA, miRNA and mRNA in both two networks was that up-regulation of lncRNA can relieve the targeted silencing of miRNA on mRNA.

For researching the interaction of mRNAs in ceRNA network, we constructed PPI networks using David online database (Fig. 5c, d). A confidence level greater than 0.4 is considered meaningful. We picked 19 and 46 mRNAs on Stromal and ESTIMATE Scores, respectively. The results revealed that in Stromal Score group, COL5A2, SPARC, THBS2, VCAM1, COL11A1 were in the center of PPI network, while LCP2, CD86, C3AR1, TLR7, PTPRC were central genes of the PPI network based on ESTIMATE Score.

### Association between LncRNA, MiRNA, MRNA in CeRNA and overall survival

**Fig. 6:**
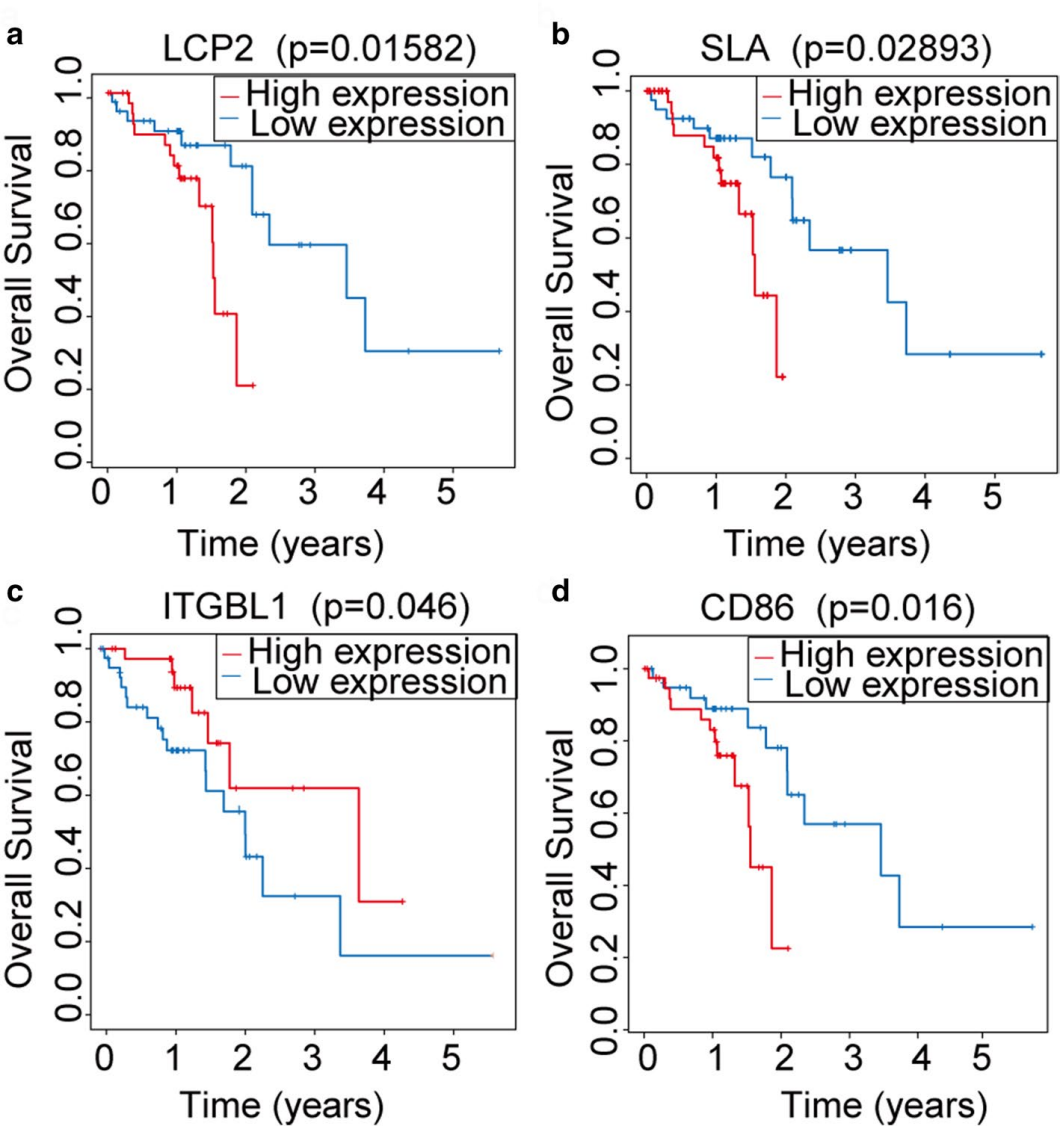
Kaplan–Meier curves with log-rank test were performed for the prognosis-related genes in ceRNA network

We analyzed the prognostic value of lncRNA, miRNA in ceRNA network and mRNA in PPI network. According to the median value of gene expression, 80 subjects were divided into high-expression and low-expression group. ITGBL1 of Stromal Score, LCP2, CD86 and SLA of ESTIMATE Score were associated with overall survival (Fig. 6).

**Fig. 7:**
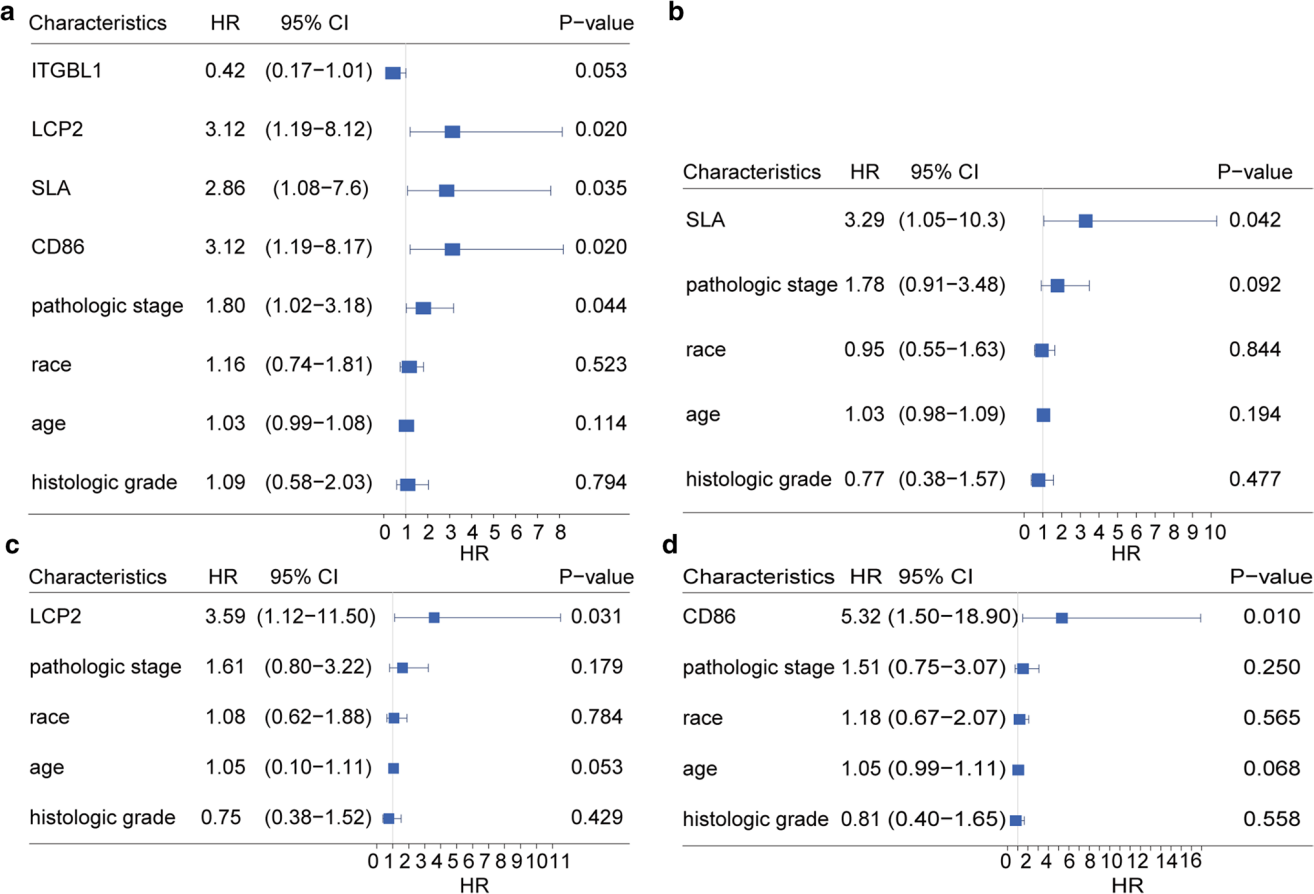
Univariate and multivariate cox proportional hazard regression analysis of three core genes

The above four mRNAs were brought into univariate cox proportional hazard regression analysis with clinical parameters (Fig. 7a), and the mRNAs with P value < 0.05 were further incorporated into multivariate analysis. Comprehensive analysis showed that LCP2, CD86 and SLA were independent prognostic factors of ESCC associated with TME (Fig. 7b–d). Low expression of these genes suggested better survival outcome.

### Survival verification of key prognostic genes

**Fig. 8:**
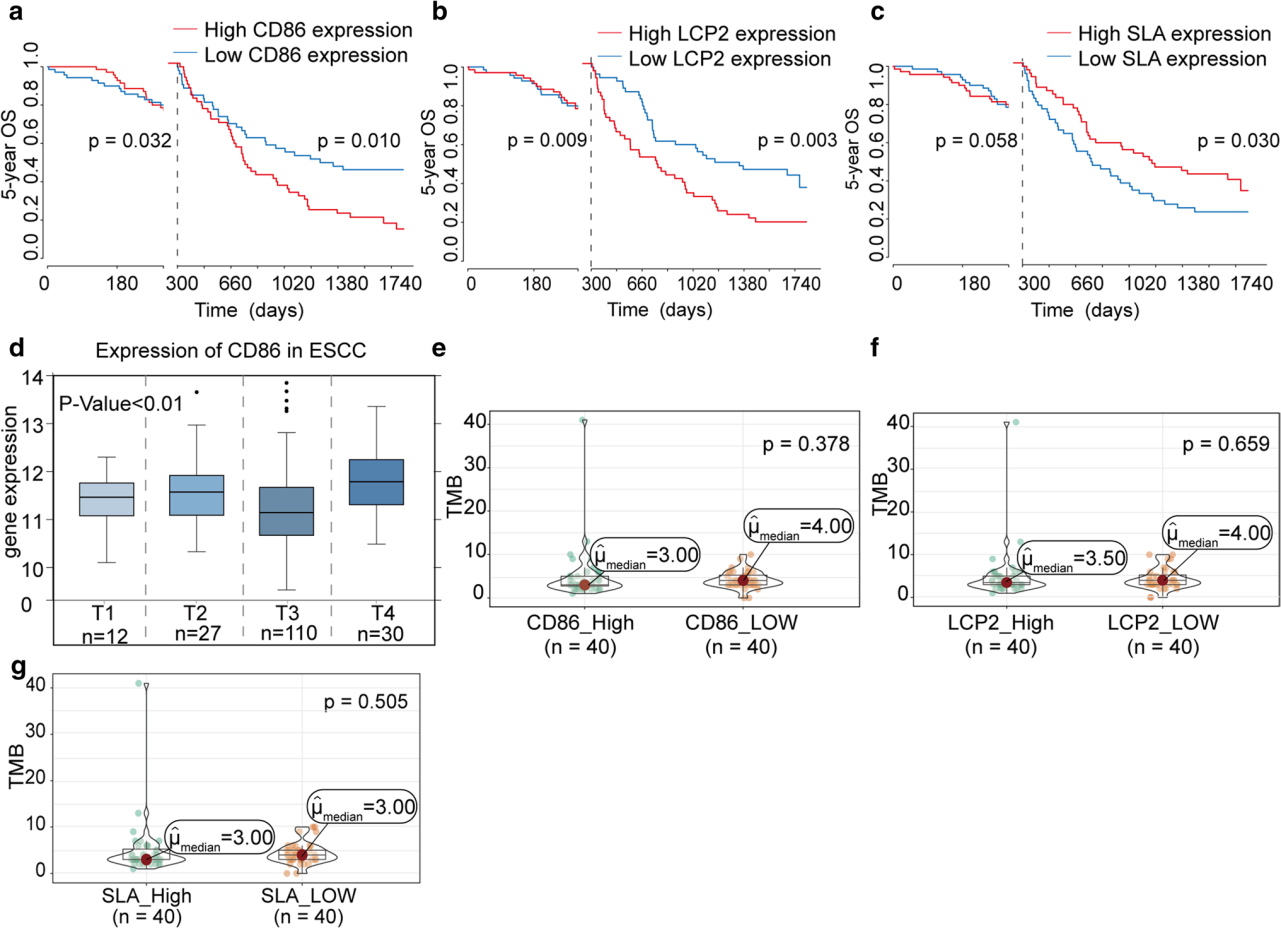
The Validation for survival and correlation analysis with TMB of three key prognostic genes. **a**–**c** Kaplan Meier curves and log rank test were performed for CD86, LCP2 and SLA in independent cohort from GEO database. **d** The correlation between the expression of CD86 and pathological stage in ESCC patients from GEO database. **e**–**g** Correlation analysis between the expression of CD86, LCP2 and SLA and TMB

Key prognostic genes were verified using 179 patients in GEO database (GSE53625). Kaplan–Meier curve indicated the significant difference between 5-year OS and expression levels of key prognostic genes (P < 0.05) (Fig. 8a–c). In TCGA and GEO databases, Low expressions of LCP2 and CD86 were related to better survival. On the Contrary to TCGA database, the 5-year OS of ESCC patients with high SLA level were significantly higher than that of patients with low SLA level. We inferred that the difference might produce by individual variations and clinical parameter diversities of these two cohorts.

Difference analysis of gene level and clinical features were performed afterwards. Kruskal–Wallis test indicated that the expression of CD86 was closely related to T stage of TNM system (P < 0.01) (Fig. 8d). The rank order of CD86 expression of ESCC T stage of TNM system was T4 > T2 > T1 > T3 (P = 0.021, P < 0.05).

Furthermore, we calculated tumor mutation burden (TMB) using comprehensive genomic profiling data from TCGA cohort and evaluated the correlation between the expression of core genes and TMB. The results showed that TMB was not significantly different between different gene expression levels (P > 0.05) (Fig. 8e–g).

### GSEA of core genes

**Fig. 9:**
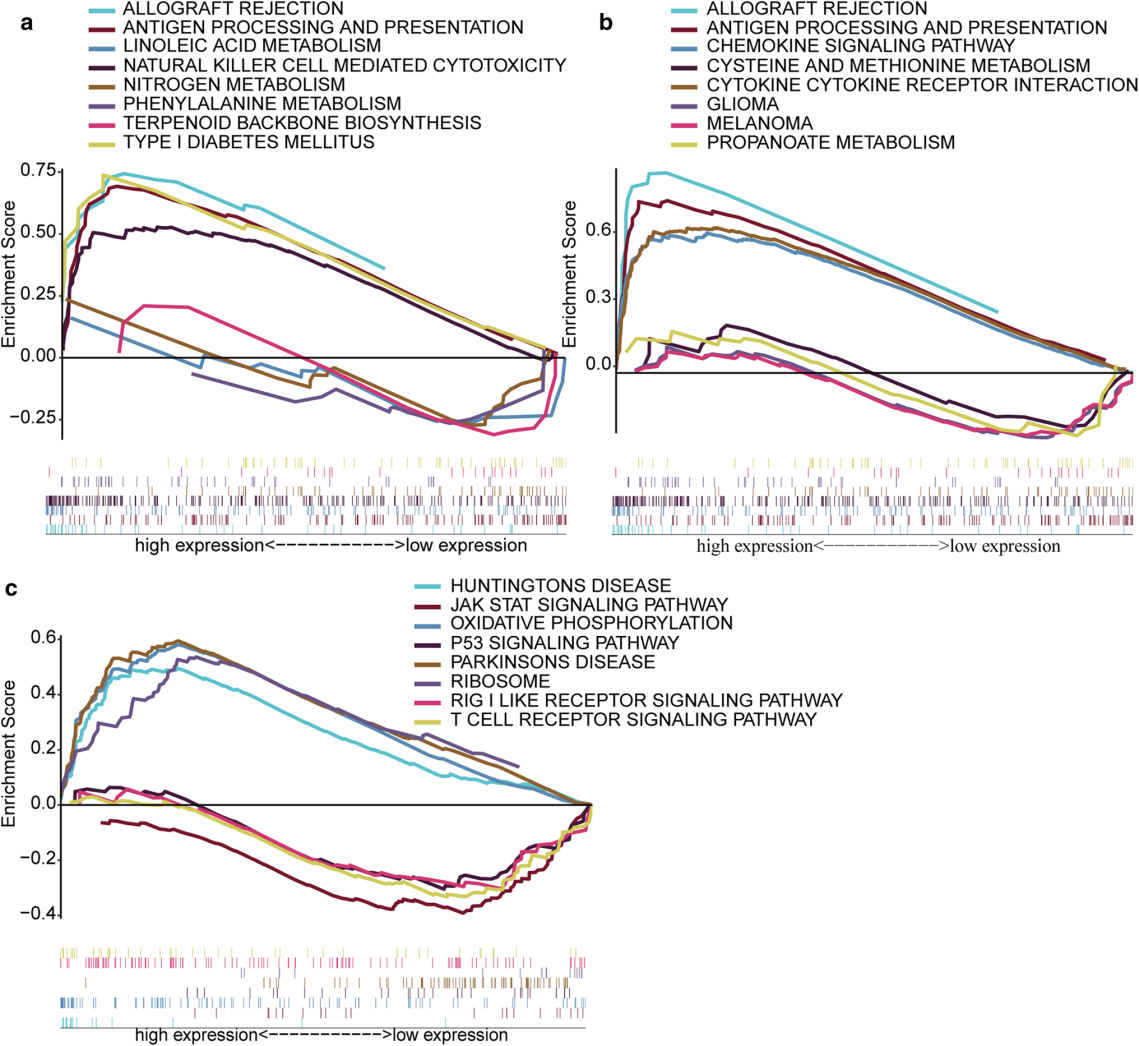
Enrichment plots from gene set enrichment analysis of key prognostic genes. **a** Signaling pathways of CD86. **b** Signaling pathways of LCP2. **c** Signaling pathways of SLA. KEGG analysis showed five signal pathways in high and low gene expression groups, respectively. Each line represented a specific gene set with unique color. The left leaning curves (positive enrichment scores) demonstrated the pathway enriched in high expression group, while the right leaning curves (negative enrichment scores) demonstrated the pathway enriched in low expression group

GSEA revealed that the genes in CD86 and LCP2 high-expression groups were mainly enriched in immune-related activities such as allografts rejection and antigen processing and presentation. As to low-expression group, the genes were enriched in metabolic pathways, including linoleic acid metabolism and cysteine and methionine metabolism (Fig. 9a, b). However, the genes in SLA low-expression group were enriched in immune-related activities including T-cell receptor signaling pathway and Rig-1 like receptor signaling pathway and tumor-related activities such as JAK-STAT signaling pathway and p53 signaling pathway. As to high-expression group, the genes were enriched in metabolic pathways such as oxidative, ribosome and phosphorylation (Fig. 9c). In ESCC patients, SLA, CD86 and LCP2 are the prognostic molecular markers of TME based on the ESTIMATE Score. GSEA result suggested that the three genes may be more inclined to immune component in TME.

### The expression of core genes is involved in immunocyte infiltration in ESCC TME

**Fig. 10:**
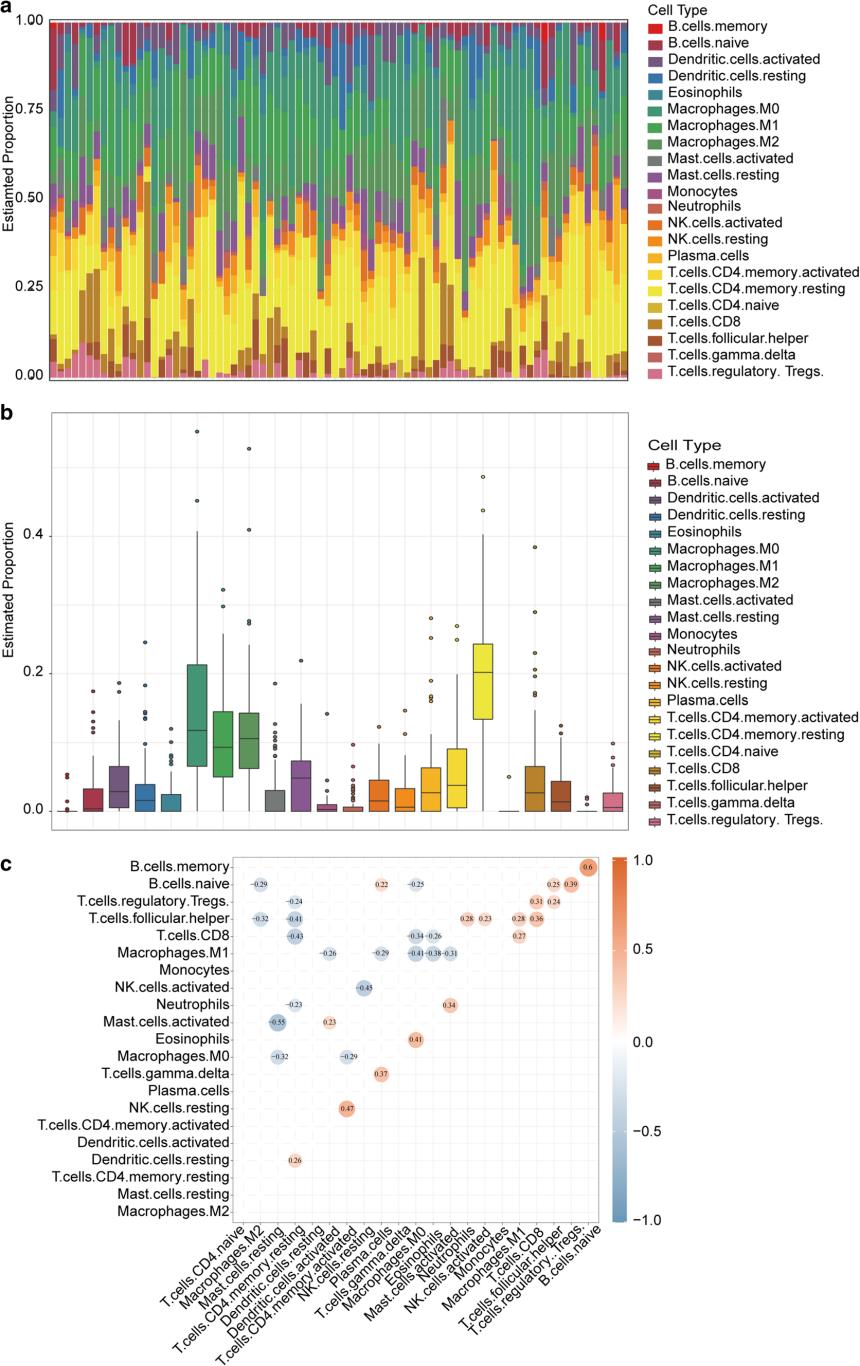
Immune cell infiltration and correlation analysis in tumor samples. **a** Scale histogram of immune cell fraction in ESCC patients. **b** Analysis of immune cell composition of 80 ESCC tumor samples. **c** The heatmap revealed the correlation between 21 kinds of TICs. The value in each small box indicates the p value of correlation between two kinds of cells. (Person analysis)

GSEA of core genes signified that these genes were mainly distributed in immune-related pathway. After estimating the average proportion of 22 immune cells in ESCC tissues, we plotted heatmap and bar graph to illustrate differential composition of immunocyte in various samples (Fig. 10a, b). Subsequently, the relevance among different immune cells was evaluated using Pearson correlation in “corrplot” software package (version 0.84) (Fig. 10c).

**Fig. 11:**
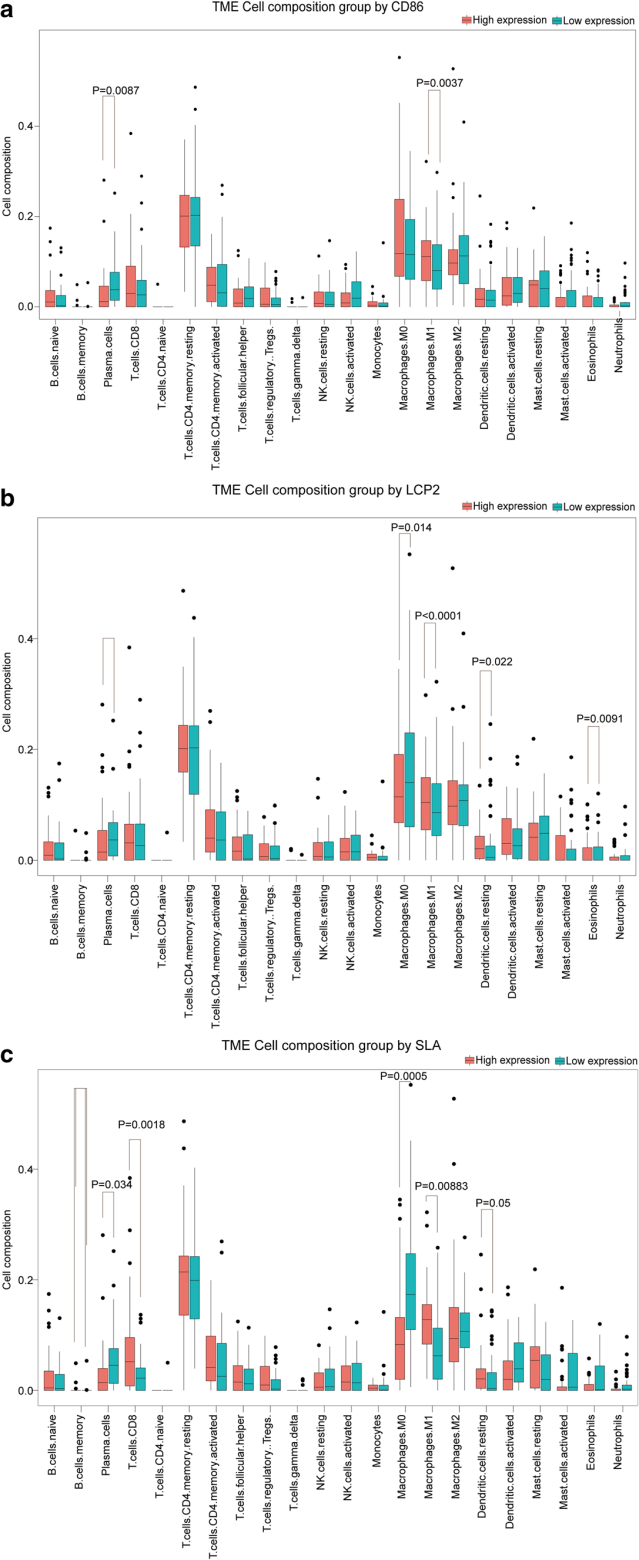
Proportions of 22 subtypes of immune cells with high and low expression of core genes. **a** Boxplot of the proportion differentiation in immune cell subtypes between high and low CD86 expression groups for ESCC. **b** Boxplot of the proportion differentiation in immune cell subtypes between high and low LCP2 expression groups for ESCC. **c** Boxplot of the proportion differentiation in immune cell subtypes between high and low SLA expression groups for ESCC

The samples were then divided into high and low groups using the median values of the three gene expressions. The difference in the proportion of immunocytes between high and low groups was computed by CIBERSORT utilizing gene expression profile. The TIC with infiltration levels of 0 in more than half of the subjects were eliminated. Wilcoxon rank sum test showed that the infiltration levels of Macrophages M1 (P = 0.0037) and Plasma cells (P = 0.0087) were affected by CD86 expression (Fig. 11a); The levels of five TICs containing Dendritic cells resting (P = 0.022), Eosinophils (P = 0.0091), Macrophages M0 (P = 0.014), Macrophages M1 (P = 0.00006) and Plasma cells (P = 0.018) vary with the expression of LCP2 (Fig. 11b); The expression of SLA affected the infiltration level of five TICs including Dendritic cells resting (P = 0.05), Macrophages M0 (P = 0.0005), Macrophages M1 (P = 0.00083), Plasma cells (P = 0.034) and T cells CD8 (P = 0.023) (Fig. 11c).

**Fig. 12:**
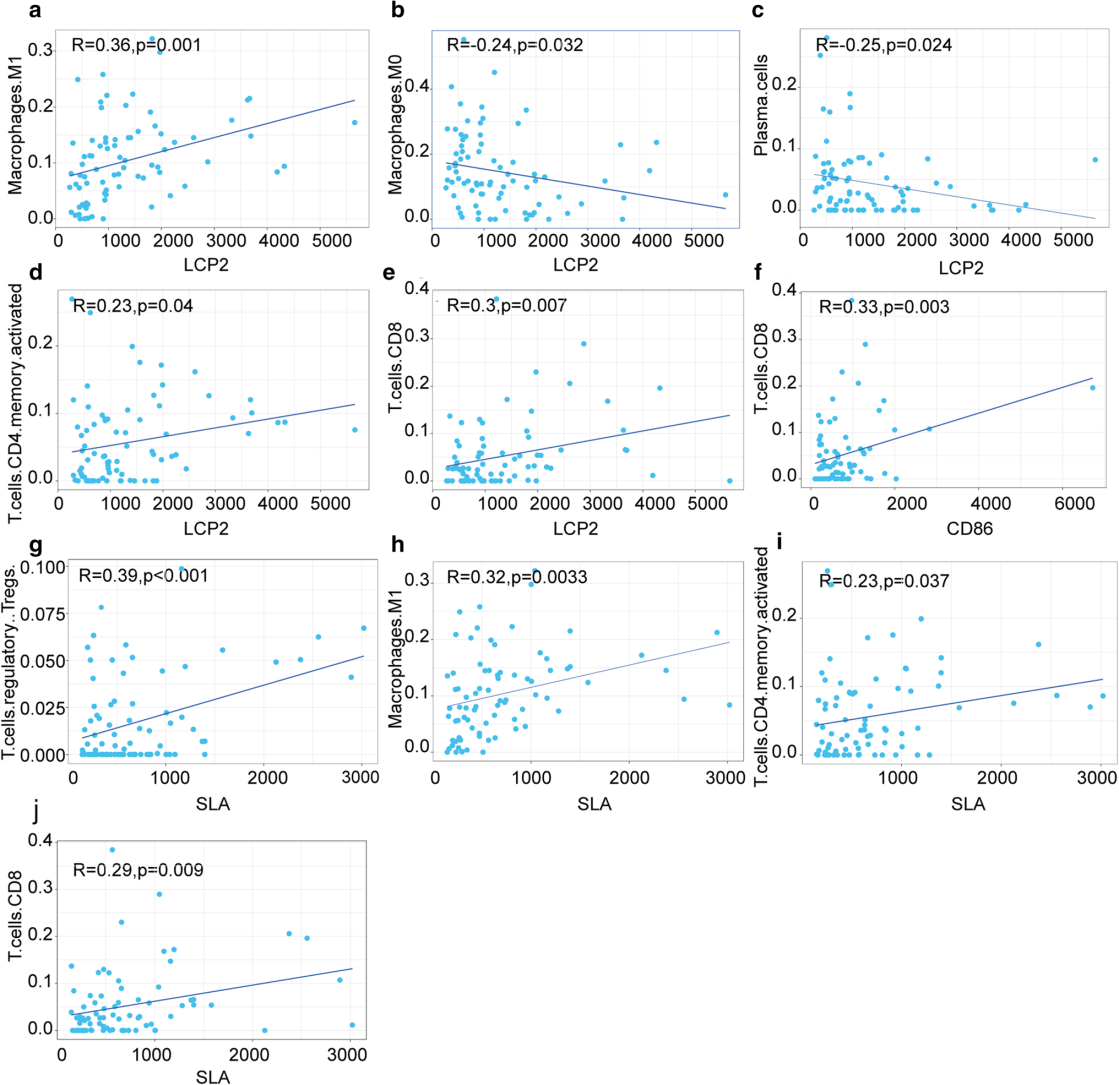
Scatter plot of the correlation between TICs proportion and gene expression (p < 0.05)

Then, we analyzed the correlation between the core genes and TIC levels (Fig. 12). Person analysis demonstrated that three kinds of TIC were positively correlated with LCP2 expression, including Macrophages M1 (r = 0.36, P = 0.001), T cells memory activated (r = 0.23, P = 0.04) and T cells CD8 (r = 0.3, P = 0.023), while two kinds of TIC such as Macrophages M0 (r = − 0.24, P = 0.032) and Plasma cells (r = − 0.25, P = 0.024) were negatively correlated with LCP2 expression; T cells CD8 (r = 0.33, P = 0.003) was positively correlated with CD86 expression; Four kinds of TIC were positively correlated with SLA expression, including T cells regulatory Tregs (r = 0.39, P < 0.001), Macrophages M1 (r = 0.32, P = 0.0033), T cells CD4 activated (r = 0.23, P = 0.037) and T cells CD8 (r = 0.29, P = 0.009). These results further confirmed that the levels of SLA, LCP2 and CD86 affect the immune activity of TME.

### IHC and H&E staining for CD86

**Fig. 13:**
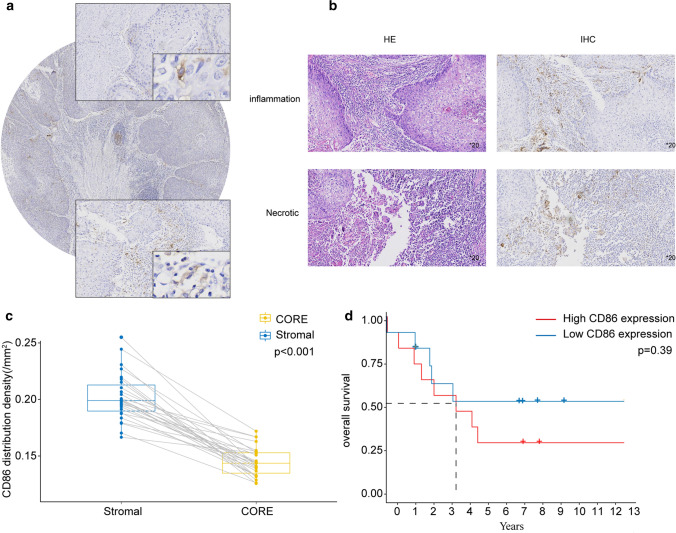
The IHC and HE Staining of 27 patients. **a** Tumor core and stromal stain of CD86 for ESCC with × 200 magnification. **b** The stain of CD86 in necrotic tissue and infiltrating inflammatory cells. **c** The two-related rank sum test of CD86 level between tumor core and stromal for ESCC patients. **d** The survival analysis between overall survival and CD86 expression

The stain of CD86 was clearly to distinguish tumor core and stromal as in shown in Fig. 13a. By two-related rank sum test, the expression of CD86 was significantly more abundant in stromal cells(P < 0.001) (Fig. 13c). A comprehensive analysis of IHC and H&E Staining indicated that CD86 was closely associated with immune-related process such as tissue necrosis and inflammatory cell infiltration (Fig. 13b). we further verify the prognostic performance of CD86 expression in ESCC. Although there was no statistical difference, the Kaplan–Meier curves demonstrated the same trend as TCGA and GEO datasets (Fig. 13d). Unfortunately, the lack of clinical information fails the validation of correlation between CD86 expression and tumor stage.

## Discussion

Over recent years, although prognosis of ESCC patients has improved to some extent with the progress of applying platinum compounds and other cytotoxic drugs into chemotherapy, the 5-year survival rate of patients is still very poor nevertheless due to the limited understanding of complex molecular mechanism in ESCC cells and the lack of more effective therapies [22]. Immunotherapy has become the hotspot in ESCC treatment. The latest research found that compared with chemotherapy, immunosuppressants such as PD-1 antibody, pembrolizumab and nivolumab can significantly improve the survival of ESCC patients [23, 24], which brings new hope for advanced patients. Despite all this, the research on ESCC tumor microenvironment and immunotherapy is still insufficient. In the presented article, we screened TME related genes based on TCGA database, and established ceRNA network by WGCNA. TME is closely associated with tumor immunotherapy. The targeted therapy for immune checkpoint inhibitors (ICI) such as PD-L1 depends on the strong infiltration of T cells in TME, however, the phenotypes of TME such as hypoxia and oxidative stress also influence the function of tumor infiltrating lymphocytes (TIL) [25].

Stromal cells and immune cells are important components of TME. In healthy tissues, stromal cells constitute the major obstacle to anti-tumor development. But transformed cancer cells can guide stromal cell reprogramming to support tumor growth and progression [26]. Furthermore, the stromal region has immunomodulatory functions, including blocking anti-tumor immunity, promoting immune escape and shaping immune cell population [27, 28]. In recent years, anti-tumor stromal cell therapy is expected to become an important clinical therapeutic measure like targeting immunotherapy. Some researches have found that the treatment aimed extracellular matrix protein (ECM), secreting by stromal cell, plays a significant role in the survival, migration and chemoresistance of ESCC [29]. In our study, by analyzing TCGA database, we also detected that the stromal score, which estimated the stromal component, is interrelated to tumor stage of ESCC patients. And the low-score group represented better survival outcome. The function of immunocytes in TME seems to be more sophisticated. On the one hand, the immune effector cells such as CD4 + T cells and CD8 + T cells were infiltrated in TME [30]; On the other hand, the existence of immunosuppressive cell groups such as Treg and exhausted CD8 T cells and the formation of immunosuppressive microenvironment may give rise to the failure of immune monitoring. It brings immunity into a tumor promoting factor [31]. The dual effect may be the major consideration for the failure that Immune Score didn’t bring discrepancies between ESCC patients’ survival in our study. Therefore, stromal cells and immune cells interact with each other and influence tumor-related immune response together. The analysis of diversity and correlation between stromal and immune cells in our study also supports the argument. We speculate that ESTIMATE Score, as the combination of Stromal and Immune Scores, is more connected to immune response. That's exactly what happened. The difference of overall survival rate in ESCC patients result from EATIMATE Score was more significant than that caused by Stromal and Immune Scores. The attack on effector immune T cells caused by immune system itself and various factors in TME may lead tumor stimulating immune response dominant in advanced patients. These possible causes may bring about the segmentation of Kaplan–Meier survival curve in our study. By constructing of PPI network, we further identified the mRNAs in the ceRNA networks. After verifying the survival value of the genes in ceRNA, we screened three independent prognostic signatures of ESCC, including SLA, CD86 and LCP2. The three genes were generated by grouping ESTIMATE Score, which means that they are related to both stromal and immune components in TME.

The GSEA of these genes disclosure that the immune response is a widely involved process. Together with the regulatory effect of stromal components on immune cells, we conjectured that the expressions of the three genes are linked with the immune cells in tumors. Consequently, we carried out TIC analysis, and the results showed that a variety of tumor infiltrating immune cells including Macrophages M1, Plasma cells and Dendritic cells resting were associated with the genes expressions.

So far, we have distinguished three TME status biomarkers related to the prognosis and clinical features of ESCC. Their possible regulatory mechanisms of ceRNA has also been predicted (MIG137HG/has-101-3p/SLA, MIG137HG/has-101-3p/LCP2 MIG137HG/has-101-3p/CD86, AF127936.3.1/has-101-3p/SLA, AF127936.3.1/has-101-3p/LCP2, AF127936.3.1/has-101-3p/CD86, AC092484.1/has-101-3p/SLA, AC092484.1/has-101-3p/LCP2, AC092484.1/has-101-3p/CD86). SLA is expressed in immune cells such as T lymphocytes and B lymphocytes [32], and negatively regulates T cell receptor signaling [33]. In our analysis of TICs, we also detected that it was connected with the expression of CD8 + , CD4 + cells. In colorectal cancer, the overexpression of SLA inhibits the tumorigenicity and invasiveness of tumor cells in colorectal cancer [34]. Our study demonstrated the action of SLA on survival for ESCC patients differs with datasets. As the research on SLA are too few to determine its specific mechanism in tumor, the phenomenon manifested in our study depends on large-scale research. LCP2 is involved in the immune process such as transmission of B cell and T cell receptor signal [35] and the activation of natural killer cell (CTL) costimulatory signa [36]. We also catch sight of the positive correlation between the expression of LCP2 and T cells in TICs analysis. LCP2 is tightly bound to the prognosis of gastric cancer [37], prostate cancer [38] and colorectal cancer [39]. In triple negative breast cancer, up-regulated expression of LCP2 recruits more immune cells and predicts a better prognosis. While in our study, the high expression of LCP2 indicated more poor prognosis for ESCC patients [40]. In addition, it is positively correlated with the expression of PD-L1, PD-1 and CTLA4, suggesting that it may be a new target for immunotherapy. CD86, the significant ligand of T cells, has a double action in immune system. It can activate T cells by binding CD28 antigen on one hand and negatively regulates T cell activation and immune response by binding the cytotoxic T-lymphocyte-associated protein 4 antigen on the other. In the published studies of esophageal cancer, CD86 is more like a positivity immune regulatory molecule. It has been found that the expression of CD86 on the surface of dendritic cells decreased in esophageal cancer TME [41, 42]. Dendritic cells (DCs) are specialized antigen presenting cells responsible for T cell activation and coordinating adaptive immunity. Immunotherapy targeting dendritic cells is a promising therapeutic measure [43]. It has been proved that dendritic cell vaccine treatment can effectively induce immune response in ESCC patients [44]. Another in vitro experiment also confirmed that nano curcumin, which has the effect of inhibiting cell proliferation, can significantly up regulate the expression of costimulatory molecule CD86 in esophageal adenocarcinoma DC [45]. In our study, the high expression of CD86 indicates a worse prognosis for ESCC patients. It is also found that CD86 had a positive regulatory effect on CD8 + T cells. The results of IHC partly authenticate these findings. Certainly, the number of validation queues is too small to verify the results of survival analysis. Furthermore, The IHC also verifies the overexpress status of CD86 in stromal cells and indicated the effect of CD86 on necrosis and inflammation. This seems to be contrary to the existing research results. We speculate that this difference may be due to the fact that most of the samples in previous studies are adenocarcinoma, and the difference of disease types may lead to different final outcomes. In the present study, using GSEA of these three genes, we also find the interesting phenomenon that these three genes may associated with the transformation of TME from immunity to metabolism. And they may also involve in the metabolism of protein, fat and other substances.

Our study also has some limitations. First of all, the samples in our study could not cover all clinical characteristics due to the failure to find a more informative and complete dataset, and the results may not be representative enough. Secondly, our study is based solely upon the bioinformatics and the validation of a small sample, and the construction of ceRNA network lacks the further experimental study on mechanism. Therefore, the biological or medical mechanisms behind identified markers are not very clear, and the wide application of the conclusion is limited as well. Prospective multi-center cohorts trials and in-depth mechanism study are needed to provide further understanding of functional roles of these molecules in the ESCC. Thirdly, we only selected CD86 for verification in the immunohistochemical. The clinical significance of the other two markers for ESCC patients requires further research.

## Conclusion

All in all, the comprehensive bioinformatics analysis was performed on the ESCC dataset in TCGA. The establishment of TME-related lncRNA-miRNA-mRNA ceRNA network was completed by WGCNA and miRNA prediction programs. In the end, TME-related gene signatures with prognostic value were screened out by TICs analysis and validation by other datasets. This study provides new alternative targets for immunotherapy of ESCC and increases the understanding of the complex interactions between ESCC tumor cells and TME.

## Supplementary Information

Additional file 1 (PDF 139 KB)

Additional file 2 (PDF 115 KB)

Additional file 3 (PDF 103 KB)

## Data Availability

Our experimental datasets are available from the corresponding authors upon reasonable request.

## Footnotes

1 https://cancergenome.nih.gov/.

2 http://www.ncbi.nlm.nih.gov/geo.

3 https://david.ncifcrf.gov/.

4 http://www.mircode.org/.

5 https://diana.e-ce.uth.gr/.

6 http://mirtarbase.cuhk.edu.cn/php/index.php.

7 http://www.mirdb.org/.

8 http://www.targetscan.org/.

9 https://string-db.org/.

10 http://www.gsea-msigdb.org/gsea/msigdb.

11 https://cibersort.stanford.edu/.

## Code availability

Our experimental datasets are available from the corresponding authors upon reasonable request.
