# Supplementary material for: Identification of prognostic biomarkers associated with tumor microenvironment in ceRNA network for esophageal squamous cell carcinoma: a bioinformatics study based on TCGA database
Source: Discov Oncol. 2021 Nov 1;12:46. doi: 10.1007/s12672-021-00442-5 (PMC8777578; doi:10.1007/s12672-021-00442-5)
Supplement: Supplementary file 1 — Additional file 1 (PDF 139 KB) [file 12672_2021_442_MOESM1_ESM.pdf]

Supplementary S1: Clinicopathological characteristics statistics of ESCC patients from TCGA

Clinical characteristics			Total (94)	%
Age at index (y)		young age (<=60)	60	63.8
		old age (>60)	34	36.2
Gender		Male	81	86.2
		Female	13	13.8
Race		Asia	45	49.5
		Black or african american	5	5
		White	41	45.5
Clinical stage	T classification	T1	5	11
		T2	12	26.7
		T3	25	55.6
		T4	3	6.7
	N classification	N0	20	55.6
		N1	14	38.9
		N2	2	5.5
		N3	0	0
	M classification	M0	41	91.1
		M1	4	8.9
Pathological stage	T classification	T1	7	7.6
		T2	31	33.7
		T3	50	54.3
		T4	4	4.3
	N classification	N0	53	58.2
		N1	29	31.9
		N2	6	6.6
		N3	3	3.3
	M classification	M0	83	95.4
		M1	4	4.6
Survival state		Alive	63	67
		Dead	31	33
Alcohol history		Yes	68	73.9
		No	24	26.1
