# Supplementary material for: Identification of prognostic biomarkers associated with tumor microenvironment in ceRNA network for esophageal squamous cell carcinoma: a bioinformatics study based on TCGA database
Source: Discov Oncol. 2021 Nov 1;12:46. doi: 10.1007/s12672-021-00442-5 (PMC8777578; doi:10.1007/s12672-021-00442-5)
Supplement: Supplementary file 2 — Additional file 2 (PDF 115 KB) [file 12672_2021_442_MOESM2_ESM.pdf]

Supplementary S2: The demographics of 27 patients.

Clinical characteristics			Total (94)	%
Age at index (y)		young age (<=60)	16	59.3
		old age (>60)	11	40.7
Gender		Male	17	63
		Female	10	37
Pathological stage	T classification	T1	3	11.1
		T2	7	25.9
		T3	17	63
	N classification	N0	22	81.5
		N1	2	7.4
		N2	2	7.4
		N3	1	3.7
	M classification	M0	27	100
Survival state		Alive	9	33.3
		Dead	13	48.2
		Unknown	5	18.5
Pathological grade		G1	10	37
		G2	10	37
		G3	7	26
