# Supplementary material for: Identification of prognostic biomarkers associated with tumor microenvironment in ceRNA network for esophageal squamous cell carcinoma: a bioinformatics study based on TCGA database
Source: Discov Oncol. 2021 Nov 1;12:46. doi: 10.1007/s12672-021-00442-5 (PMC8777578; doi:10.1007/s12672-021-00442-5)
Supplement: Supplementary file 3 — Additional file 3 (PDF 103 KB) [file 12672_2021_442_MOESM3_ESM.pdf]

Supplementary S3: Immunohistochemistry (IHC) and Hematoxylin-eosin (HE) staining

Hematoxylin-eosin (HE) staining

Cut the paraffin-embedded esophagus tissue into thin slices. Then, they were dewaxed by xylene (3 times,3 min respectively) and hydrated by graded alcohol (100%,85%,75%, 3min respectively). After rinses with running water, hematoxylin solution was used to stain (5 min). Then, the slides were dehydrated again with ethanol and followed by wash with running water (5 min). Finally, the slides were stained again with eosin. Sealed the slides after dehydration.

Immunohistochemistry (IHC)

Paraffin-embedded esophagus tissue sections were placed in a 70C oven for 60 min. The slides were deparaffinized in xylene and then hydrated by graded alcohol (100%, 85%, 75%, 5min respectively). Put the slides in 100 C citrate solution for 4min to repair antigen (high pressure) and next rinsed them with running water until cool down to room temperature. Then, the slides were washed 3 times with PBS for 3min each time. After an incubation with endogenous peroxidase blocker for 15min at room temperature (Maixin, China) and washed with PBS, the sections were incubated with normal nonimmune serum to block nonspecific staining (15min, room temperature). Then, all slides were incubated with anti-CD86(91882S, 1:200, CST, America) overnight at 4 C. Thawed the slides for 1 h at room temperature and followed wash with PBS (3 times, 3min respectively). The samples were incubated with secondary antibody (15min, RT) followed by PBS rinses (3 times, 3min respectively). After an incubation with streptavidin peroxidase complex (Maixin, China) and PBS rinses (3 times, 3min respectively), the diaminobenzidine (DAB) was used to stain tissue samples (3 min and 30s). Finally brief hematoxylin staining (1min and 30s) was performed after washing with running water(5min). Sealed the slides with resin after dehydration.
